# Inhibition of interleukin-1 or -6 after myocardial infarction: pros, cons, and future perspectives

**DOI:** 10.1093/ehjcvp/pvaf022

**Published:** 2025-03-29

**Authors:** Mattia Galli, Filippo Luca Gurgoglione, Sebastiano Sciarretta, Antonio Abbate

**Affiliations:** Department of Medical–Surgical Sciences and Biotechnologies, Sapienza University of Rome, Corso della Repubblica 79, Latina 04100, Italy; Maria Cecilia Hospital, GVM Care & Research, Via Corriera 1, 48033 Cotignola, Italy; Division of Cardiology, Parma University Hospital, University of Parma, Parma, 43126, Italy; Department of Medical–Surgical Sciences and Biotechnologies, Sapienza University of Rome, Corso della Repubblica 79, Latina 04100, Italy; Department of AngioCardioNeurology, Istituto di Ricovero e Cura a Carattere Scientifico Neuromed, Pozzilli, 86077, Italy; Division of Cardiology and Berne Cardiovascular Research Center, University of Virginia, Charlottesville, VA 22903, USA

Myocardial infarction (MI) results from atherosclerotic plaque rupture and thrombus formation, triggering a sustained inflammatory response. Interleukin-1 (IL-1) and IL-6 are key mediators driving local inflammation and myeloid cell recruitment, contributing to impaired healing, adverse remodelling, and poor outcomes.^1^ On this background, IL-1 receptor antagonists have been tested as therapeutic targets in patients with MI. In a large phase III trial, canakinumab, an IL-1β blocking antibody, has demonstrated a 15% reduction in the risk of major adverse cardiovascular events (MACE) at 4 years compared with placebo, with a direct correlation between the lowering of IL-1β and the decreased risk of MACE.^2^ Despite these promising results, the premature interruption of the clinical programme development of canakinumab by Novartis dampened the enthusiasm for IL-1β as a target. Nevertheless, anakinra, a recombinant IL-1 receptor antagonist, has also been evaluated in three phase II studies. A pooled analysis of 139 patients with ST-segment elevation MI (STEMI) demonstrated a reduction in the composite endpoint of new-onset heart failure and death compared with placebo.^3^ More recently, another IL-1 blocker, goflikicept, has also shown to reduce systemic inflammation in patients with STEMI.^4^

On the other hand, ziltivekimab, a monoclonal antibody directed against IL-6, downstream of IL-1, has shown to reduce inflammatory markers among patients with chronic kidney disease with a rather favourable safety profile.^5^ The ongoing phase IIIa ARTEMIS trial will determine whether ziltivekimab can effectively reduce the risk of MACE in patients with MI. Additional phase III studies with ziltivekimab are also ongoing in stable cardiovascular and kidney disease (ZEUS trial) and in heart failure with preserved or mildly reduced ejection fraction (HERMES trial).
